# Children’s Shyness, Frontal Brain Activity, and Anxiety in the Perioperative Context

**DOI:** 10.3390/bs13090766

**Published:** 2023-09-14

**Authors:** Cheryl H. T. Chow, Kristie L. Poole, Richard Y. Xu, Jhanahan Sriranjan, Ryan J. Van Lieshout, Norman Buckley, Graeme Moffat, Louis A. Schmidt

**Affiliations:** 1 Department of Psychology, Neuroscience & Behaviour, McMaster University, Hamilton, ON L8S 4L8, Canada; poolekristie@gmail.com (K.L.P.); richard.xu@medportal.ca (R.Y.X.); jhanahan.sriranjan@medportal.ca (J.S.); schmidtl@mcmaster.ca (L.A.S.); 2 Michael G. DeGroote School of Medicine, McMaster University, Hamilton, ON L8S 4L8, Canada; 3 Department of Psychiatry & Behavioural Neurosciences, McMaster University, Hamilton, ON L8S 4L8, Canada; vanlierj@mcmaster.ca; 4 Department of Anesthesia, McMaster University, Hamilton, ON L8S 4L8, Canada; buckleyn@mcmaster.ca; 5 InteraXon Inc., Toronto, ON M5V 3B1, Canada; graeme.moffat@gmail.com

**Keywords:** preoperative anxiety, child, shyness, electroencephalography, temperament, surgery

## Abstract

Although preoperative anxiety affects up to 75% of children undergoing surgery each year and is associated with many adverse outcomes, we know relatively little about individual differences in how children respond to impending surgery. We examined whether patterns of anterior brain electrical activity (i.e., a neural correlate of anxious arousal) moderated the relation between children’s shyness and preoperative anxiety on the day of surgery in 70 children (36 girls, *M*_age_ = 10.4 years, *SD*_age_ = 1.7, years, range 8 to 13 years) undergoing elective surgery. Shyness was assessed using self-report approximately 1 week prior to surgery during a preoperative visit (Time 1), preoperative anxiety was assessed using self-report, and regional EEG (left and right frontal and temporal sites) was assessed using a dry sensory EEG headband on the day of surgery (Time 2). We found that overall frontal EEG alpha power moderated the relation between shyness and self-reported preoperative anxiety. Shyness was related to higher levels of self-reported anxiety on the day of surgery for children with lower average overall frontal alpha EEG power (i.e., higher cortical activity) but not for children with higher average overall frontal alpha EEG power (i.e., lower cortical activity). These results suggest that the pattern of frontal brain activity might amplify some shy children’s affective responses to impending surgery. Findings also extend prior results linking children’s shyness, frontal brain activity, and anxiety observed in the laboratory to a real-world, ecologically salient environment.

## 1. Introduction

One of the most common stressful experiences a child can encounter is the anticipation of surgery. Preoperative anxiety affects up to 75% of the 6 million children who undergo surgery in North America each year [1]. It is associated with adverse clinical (e.g., increased pain and recovery time), psychological (e.g., separation anxiety), and behavioural outcomes (e.g., aggression towards authority figures) [2] that can have significant short- and long-term implications for health and functioning. Children with preoperative anxiety are at increased risk for nightmares, feeding and sleeping problems, and enuresis [3,4]. Given the negative impact preoperative anxiety can have on children and families, researchers have begun to focus their attention on understanding the etiology of this phenomenon as a means of identifying targets for intervention. The ultimate goal of this work is to minimize the prevalence of preoperative anxiety, its severity, and its adverse effects.

### 1.1. Children’s Temperament and Preoperative Anxiety

Relatively few studies have examined whether individual differences in children’s temperament might help to predict preoperative anxiety. Kain and colleagues [5] showed that poor social adaptability and an inhibited, shy temperament were independent predictors of increased preoperative anxiety. In another study, children who scored low on sociability (i.e., who were shy) also exhibited increased levels of perioperative anxiety from immediately before the surgery to 2 weeks postoperatively [6]. A previous pilot study also showed that, paradoxically, temperamental shyness was inversely related to preoperative anxiety for school-aged children undergoing elective surgery [7]. This discrepancy may have been due in part to methodological differences between studies, such as small sample sizes and method variance. A recent systematic review and meta-analysis conducted by Chow et al. [8] showed that certain temperament styles, such as emotionality, intensity of reaction, and withdrawal, were associated with increased preoperative anxiety, whereas activity level was associated with reduced anxiety. However, relatively little is known about whether individual differences in shyness are consistently associated with perioperative anxiety.

### 1.2. Children’s Shyness and Anxiety

Outside of the surgical setting, individual differences in temperament have been shown to serve as vulnerability factors for the later development of anxiety (see [9] for a review). For example, temperamental shyness (i.e., a preoccupation with the self in response to real or imagined social interactions) has been consistently linked to an increased risk for anxiety and depression in typically developing children [10]. In general, children who are rated as temperamentally shy have a wide range of social and emotional problems (e.g., emotion dysregulation, poor peer relations, and social rejection) [11]. Moreover, temperamental shyness and anxiety-related symptoms are linked across development and have been noted in children (see [12,13,14] for reviews). However, not all shy children are the same in their display of anxiety, raising the possibility of moderating factors. One potential moderator factor that might help us understand the relation between shyness and anxiety is individual differences in anterior brain activity.

### 1.3. Overall Frontal Brain Activity and Emotional Experience

Several investigators [15,16,17] have argued that the pattern of overall frontal EEG alpha power may track and reflect the intensity of affective experience. Given the inverse relation between EEG alpha power and cortical activity, relatively lower EEG alpha power reflects relatively higher cortical activity [18]. Thus, relatively lower EEG alpha power has been inferred to reflect greater emotional arousal or intensity in studies of emotion.

For example, Dawson and her colleagues [19] reported that infants exhibited a decrease in overall frontal EEG power (i.e., ostensibly more emotional arousal) during maternal separation, and this was paralleled by more behavioural signs of distress. Still, another study conducted on adults found that overall frontal EEG alpha power characterized emotional experiences in people who were shy [20]. Those who were shy exhibited relatively lower overall frontal alpha power (i.e., more cortical activity) in anticipation of a social encounter compared with less shy adults. This raises the possibility that overall frontal EEG alpha power might reflect an endophenotype linking shyness and emotional experience.

Moreover, contrasting EEG patterns of alpha activity have been shown to distinguish two types of anxiety (i.e., anxious arousal and apprehension) in adults, with anxious arousal exhibiting more right than left activity and anxious apprehension showing more bilateral activity [21,22,23]. Several studies have reported an increase in bilateral hemispheric activity reflecting anxious apprehension [22,23] or the co-occurrence of both anxious arousal and anxious apprehension [24] during intense emotional experiences. In particular, extremely anxious apprehension could make an individual more vulnerable to experiencing anxious arousal simultaneously when confronted with stressful events [23].

The anticipation of an impending surgery is a powerful stressor that can elicit negative emotions (i.e., crying, sadness, fear) and anxiety for children undergoing surgery. One recent pilot study conducted by our group has demonstrated that higher frontal brain activity during preoperative visits was correlated with higher self-reported state anxiety on the day of surgery [25]. Given that previous research has established associations between shyness and anxiety outside of a surgical context and that overall frontal activity is related to emotional experience, it is possible that the pattern of overall frontal brain activity in response to stress (i.e., surgery) may track emotional experience and moderate the relation between individual differences in temperament and anxiety.

To our knowledge, only one study has examined the neural correlates of temperament and anxiety in children in an ecologically salient environment such as the surgical setting [26]. The preoperative setting and time to surgery provide a novel, real-world environment to study fear-related behaviours.

### 1.4. The Present Study

In this study, we collected study measures on two separate occasions. During a preoperative visit, which occurred 7 to 10 days prior to surgery (Time 1; T1), children self-reported their level of shyness. On the day of the surgery visit (Time 2; T2), we collected 40 min of continuous EEG from the child using the Muse headband, and children also self-reported their level of preoperative anxiety.

The goal of the present study was to examine whether average overall frontal EEG alpha power at T2 moderated the relation between children’s self-reported shyness at T1 and self-reported state anxiety at T2. Based on previously published work [25,26], we predicted the average overall frontal brain activity would moderate the relation between shyness and preoperative anxiety, such that shyness would be related to higher levels of self-reported anxiety on the day of surgery for children with lower average overall frontal alpha EEG power (i.e., higher cortical activity; a marker of anxious arousal), but not for children with higher average overall frontal alpha EEG power (i.e., lower cortical activity). We also examined whether average overall temporal EEG alpha power also moderated the relation between shyness and preoperative anxiety to test the hypothesis that the effects were specific to the frontal region.

## 2. Method

### 2.1. Participants

Table 1 shows the demographic characteristics of the children and their parents in this study. Seventy children (ages 8 to 13 years) who were scheduled to receive elective outpatient surgery (e.g., tonsillectomy and adenoidectomy) and their parents were included in this study. Children and families in this study were recruited as part of a larger study examining the management of children’s preoperative anxiety. Recruitment took place during the child’s preoperative clinic visit 7–10 days before surgery at McMaster University Medical Centre’s affiliated Children’s Hospital located in Hamilton, Ontario. Children diagnosed with significant neurodevelopmental disorders (e.g., organic brain disorders (i.e., brain injury), neurosensory impairment (i.e., visual and/or hearing impairments), etc.) and families who were unable to provide assent and consent, or who could not understand or speak English, were not eligible to participate.

### 2.2. Procedure

Eligible children were approached during their preoperative visit (T1) at the McMaster Children’s Hospital and were invited to participate in the study. During this visit, children and families met with a child life specialist (someone who provides emotional support to help prepare children and families for their surgery), a pediatric nurse, and an anesthetist as part of standard care at the hospital. After obtaining parental consent and child assent, demographic information and a measure of the child’s temperament were collected.

On the day of surgery (T2), children and families were met by a research assistant in the surgical waiting area. Children and families were prepared for the surgery by a pediatric nurse, and then children and their parent(s) were moved to a holding area outside of the operating room (OR), where EEG data were collected, and self-reported child anxiety was measured. A $25 gift card was given to all participating families as a token of appreciation for their participation in the study. All procedures were approved by the Hamilton Integrated Research Ethics Board (HiREB).

### 2.3. Temperament and Preoperative Anxiety Measures 

**Child-Report of Shyness**. Shyness was measured using the five highest-loaded items [27] from the original Cheek and Buss Shyness Scale [28,29] to reduce participant burden. A sample item from this scale includes “I find it hard to talk to strangers”. Children responded on a five-point Likert scale ranging from 0 (not at all characteristic) to 4 (extremely characteristic), and so higher values indicated higher shyness. A mean shyness score was computed using the five items. This scale demonstrated good internal consistency in our sample (α = 0.80). Children’s shyness was assessed at the preoperative visit, T1.

**Children’s Perioperative Multidimensional Anxiety Scale** (CPMAS). The CPMAS is a validated self-report scale designed to measure children’s state anxiety within a surgical context [30]. The CPMAS contains five items, to which the child responds on a visual analog scale (i.e., each item ranging from 0 (Not worried at all) to 100 (Very worried)). The CPMAS was found to have very good internal consistency (α = 0.93) in the present study. Children’s anxiety was assessed immediately before surgery, T2.

### 2.4. EEG Data Recording, Reduction, and Analysis

**EEG Data Recording and Collection**. EEG data were collected with children’s eyes open for approximately 40 min while the child was seated waiting for their starting surgery after nurse preparation up until anesthetic induction, T2. EEG data were recorded using a Muse headband (InteraXon Inc., Toronto, ON, Canada). The Muse headband is a wireless quantitative EEG headband equipped with four dry sensor channels (i.e., frontal sites (AF7, AF8) and temporal sites (TP9, TP10)) referenced to a fifth channel located at the medial prefrontal region (Fpz). The Muse headband was paired to an Android tablet (Samsung Galaxy Tab A; Samsung Electronics Co., Ltd., Suwon, Republic of South Korea) via Bluetooth. During the acquisition phase, the Mind Monitor application (an interface software) was used to capture and record data. The raw EEG signals were sampled at 256 Hz. The Muse headband has demonstrated reliability with other existing portable and laboratory-based (i.e., “wet”) EEG systems (see ref. [31] for comparison of EEG systems) and demonstrated good psychometric properties in the preoperative setting [25].

**EEG Data Reduction and Analysis**. A real-time fast Fourier transform (FFT) was employed by the Muse Monitor application to record and compute the power spectral density in the alpha frequency band (7.5 to 13 Hz). We examined EEG power in the alpha frequency range because this frequency band has been consistently linked to individual differences in affective style and anxiety (see [32] for a recent review). A notch filter was set at 60 Hz to reduce artifacts and noise, and additional noise suppression was applied using the Muse’s built-in driven right leg circuit (with a 2 µV noise floor) for all channels. Visual inspection was performed to remove artifacts emerging from signal connectivity issues. The onset and offset time were marked, and those data were eliminated from analyses.

A composite measure of average overall alpha power was computed separately for the frontal and temporal sites by averaging overall alpha power separately across the two frontal sites (i.e., Time 2 (AF7 + AF8)/2) and two temporal sites (i.e., Time 2 (TP9 + TP10)/2)). We collected measures in the temporal region to ensure the effects were specific to the frontal region. Because EEG alpha power is inversely related to cortical activity, lower scores on the overall frontal and temporal metrics reflect more brain activity [18,33,34].

### 2.5. Data Loss

Due to unforeseen surgery scheduling conflicts (e.g., appointment cancellations, deviations from schedule), a few children were unable to complete all the measures across both visits and were excluded from analyses. All 70 children had self-reported temperament data, 60 had useable EEG data at T2, and 66 had self-reported anxiety data at T2. Across the measures, a total of 59 had complete data and are included in the regression analyses below.

### 2.6. Statistical Analysis

Descriptive analyses were conducted to summarize the baseline characteristics of the children and families in this study. A series of chi-square and independent samples *t*-tests were performed to examine differences in participant sex, age, handedness, and previous hospitalizations. A series of Pearson correlations were also performed to examine relations among the children’s shyness, overall frontal and temporal EEG alpha power, and preoperative anxiety. We used a multiple linear regression model to examine if frontal alpha power at T2 moderated the relation between shyness at T1 and self-reported anxiety at T2. In the first step, we entered shyness; in the second step, we added frontal alpha power; and in the final step, we entered an interaction term that was the cross-product of shyness and frontal alpha power. We also performed identical analyses using average overall temporal alpha power as a moderator to test for specificity between the frontal and temporal brain regions.

All statistical analyses were performed using SPSS Version 24.0, with significance levels set at α = 0.05.

## 3. Results

### 3.1. Descriptive Statistics

Participant sex, age, handedness, and previous hospitalizations were not related to shyness, overall frontal and temporal EEG alpha power, or preoperative anxiety (*p*s > 0.05). Intercorrelations, means, and standard deviations (*SD*) for study variables are shown in Table 2.

### 3.2. Regression Analysis

**Figure 1:**
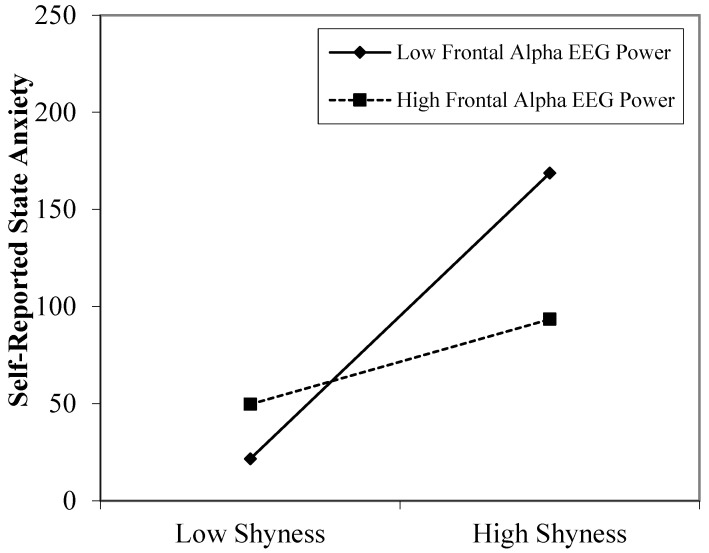
Significant interaction effect of overall frontal alpha power on the relation between individual differences in shyness and state anxiety during the day of elective surgery in children. EEG power is inversely related to activity, so lower overall EEG power reflects greater overall activity. Values are plotted at one standard deviation above and below the mean.

The final model was significant in predicting self-reported state anxiety, *F*(3, 55) = 7.92, *p* < 0.001, *R*^2^ = 0.30. As presented in Table 3, results revealed that average overall frontal alpha EEG power moderated the relation between self-reported shyness and self-reported state anxiety, *p* = 0.019. To probe this interaction, we examined the influence of shyness on self-reported anxiety at high (1 standard deviation above the mean) and low (1 standard deviation below the mean) levels of frontal alpha EEG power. As illustrated in Figure 1, simple slope analyses revealed that shyness was positively related to state anxiety in children with low levels of average overall frontal alpha power (β = 80.87; *p* < 0.001), but shyness was unrelated to state anxiety in children with high levels of average overall frontal alpha power (β = 10.67; *p* = 0.619).

We repeated the above analyses using average overall temporal alpha EEG power for sensitivity analyses. In the first step, shyness was entered and was a significant predictor of self-reported state anxiety (β = 51.98; *p* < 0.001). In the second step, average overall temporal alpha EEG power was not a significant predictor of self-reported state anxiety (β = −60.04; *p* = 0.625). Finally, in the third step, the interaction term was not significant (β = −155.25; *p* = 0.206), indicating that average overall temporal alpha power did not moderate the relation between shyness and state anxiety and specificity to the frontal region.

## 4. Discussion

In this study, we explored relations among self-reported shyness, average overall anterior EEG alpha power, and self-reported preoperative anxiety in school-age children undergoing elective surgery. Our findings revealed that the average overall frontal EEG alpha power measured on the day of surgery moderated the association between shyness measured during a preoperative visit and preoperative anxiety measured during the day of surgery. We found no such links for temporal brain activity.

The present study extends the existing literature concerning the role of individual differences in temperament in childhood anxiety. Our findings are consistent with previous work that has been conducted outside of the surgical context in children, where shyness has been linked to increased levels of anxiety (e.g., [12,13]; see also [14] for a review). Here, we extend these findings by demonstrating similar associations but within a “real world” ecologically salient context (e.g., surgery).

When compared to work that has been carried out within the surgical context, our study was consistent with prior research that showed that children who had an inhibited temperament (i.e., were shy) experienced higher preoperative anxiety [2] (see also [8] for a recent review). However, our findings were not consistent with the results of a preliminary study carried out by Chow and colleagues [7], where shyness was found to be negatively correlated with preoperative anxiety for school-aged children undergoing elective surgery. This discrepancy may have been due to some methodological differences between the two studies. For example, the proportion of children who had previously been hospitalized in the present study (50%) was greater than in the previous study (29%). It is possible that children who had undergone prior surgical experience(s) might react differently than those who had never been in the preoperative environment. However, the field examining relations between temperament and anxiety in ecologically valid, non-normative contexts is relatively new. Therefore, very little is known about the types of normative behaviours that children exhibit within this context. We postulated that other inhibited temperamental styles may also be associated with anxiety in children undergoing surgery. More research needs to be conducted to examine the possible confounding factors (e.g., other temperamental styles, previous hospitalization, types of preparation, types of stressors, and parenting styles) that could directly and indirectly affect children’s behaviours in this unique context for studying children’s anxiety.

To further our understanding of the biologically based factors underlying links between shyness and preoperative anxiety, we explored the role of overall frontal EEG alpha power on this relation. Our results suggest that average overall frontal brain power may track preoperative anxiety in shy children in the surgical setting. Consistent with the literature [15,16,20], the pattern of overall frontal alpha brain activity (i.e., the inverse of EEG alpha power) may reflect the intensity of emotional experience during the perioperative period, with relatively higher overall frontal activity reflecting a more intense emotional experience for children who were shy than relatively lower overall frontal brain activity. Furthermore, the elevated overall frontal bilateral EEG activity may track emotional arousal (i.e., anxious apprehension) that is experienced by temperamentally shy children leading up to surgery. This heightened arousal may reflect the hyperactivity of the underlying cortical processes within the prefrontal cortex and related subcortical structures [35].

### Limitations

The following limitations should be noted when interpreting the findings of the present study. First, although this study is believed to represent one of the first of its kind (i.e., collecting EEG data in real time) in relation to children’s temperament and anxiety in the preoperative context, and given its relatively small sample size and correlational design, our results need to be replicated with a larger sample before causal inferences can be made. Second, although well-validated self-report measures were used to assess temperament and anxiety, these measures may be subject to some bias as they were self-reported. Other validated objective measures, such as behavioural measures of temperament and physiological indicators of stress (e.g., heart rate, cortisol), could be included in future work in order to more completely characterize the phenomena of temperament and preoperative anxiety in perhaps a more objective and less biased way. In addition, obtaining more data throughout the entire perioperative period (e.g., EEG signals during and after surgery and outside of the surgical context) might also provide further insights into the course of perioperative anxiety and its influence on recovery. Third, while the portable EEG system Muse allows for capturing continuous real-time data non-invasively in the surgical context, only a limited number of recording channels were available. Therefore, the study needs to be replicated in other contexts (e.g., home and the laboratory) using both traditional and more extensive EEG systems to ensure the reliability of the present results. Finally, to maintain a relatively homogenous sample, we restricted our sample to school-age children receiving elective surgery only. This does impose some limitations on the generalizability of our results to all children undergoing surgery (e.g., non-elective), and so future studies should examine the link between temperament and preoperative anxiety across a broader range of developmental stages and elective and non-elective surgical procedures. Finally, future studies should also include a follow up after surgery to measure potential short- and long-term adverse postoperative outcomes (e.g., postoperative behaviour, anxiety, and pain).

## 5. Conclusions

The findings of this study extend prior work by examining paths linking shyness, frontal brain activity, and anxiety in other stressful contexts to a real-world environment. We believe that this novel work has both theoretical (i.e., understanding the relation between temperament and anxiety) and clinical (i.e., understanding the development and management of preoperative anxiety) implications. On a theoretical level, the findings of the present study extend the current literature on the link between shyness and anxiety by examining these phenomena in other contexts. Our results also provided support for the observation that average overall frontal EEG activity moderated the relation between shyness and preoperative anxiety, providing insight into individual differences and why not all shy children react the same way to impending surgery. Clinically, this preliminary investigation sheds light on the etiology of preoperative anxiety and may help guide future research in the understanding of the biological correlates underlying the links between temperament and anxiety in stressful contexts. Given the prevalence and negative effects of preoperative anxiety, the ultimate goal is to help guide and personalize the future design of preventive and intervention strategies, such as the development of better coping methods in managing preoperative anxiety for children undergoing surgery, specifically for children who are shy, that might target a change in patterns of anterior brain physiology.

## Figures and Tables

**Table 1:** Demographic characteristics of children and families in the study.

Sample	Characteristics	
		*n* = 70
Children		
	Sex, *n* (Boys/Girls)	34/36
	Age, *M* ± *SD*	10.4 ± 1.7
	Child life specialist preparation, *n* (Yes/No/unknown)	58/4/8
	Previous hospitalization, *n* (Yes/No)	32/38
Parents		
	Mother/Father/Other *, *n* (Mother/Father/Other)	56/10/4
	Age (in years), *M* ± *SD*	41.0 ± 5.8

*M* = Mean; *SD* = Standard Deviation. * = Grandparent/Stepparent.

**Table 2:** Intercorrelations, means, and standard deviations (SD) for study variables.

Measure	1	2	3	4	Mean (SD)
1. Child-reported shyness T1	-	0.44 **	−0.07	−0.07	1.77 (1.02)
2. Self-reported anxiety T2		-	−0.25	−0.09	152.26 (121.83)
3. Overall frontal alpha power T2			-	0.70 **	0.58 (0.15)
4. Overall temporal alpha power T2				-	0.89 (0.12)

SD = standard deviation; T1 = preoperative visit; T2 = day of surgery visit, ** *p* < 0.001; 1 = Child-reported shyness T1; 2 = Self-reported anxiety T2; 3 = Overall frontal alpha power T2; 4 = Overall temporal alpha power T2.

**Table 3:** Summary of regression model of shyness and overall frontal brain activity predicting self-reported state anxiety on day of elective surgery in children.

		Self-Reported State Anxiety				
	Predictors	Beta	*S.E.*	*p*-Value	R^2^	ΔR^2^
Step 1					0.180	0.180 *
	Shyness	51.98	14.67	0.001		
Step 2					0.227	0.047
	Shyness	49.96	14.42	0.001		
	Frontal Alpha EEG Power	−179.881	97.78	0.071		
Step 3					0.302	0.075 *
	Shyness	179.18	55.07	0.002		
	Frontal Alpha EEG Power	282.49	212.56	0.189		
	Shyness X Frontal Alpha EEG Power	−232.83	96.05	0.019		

* *p* < 0.05.

## Data Availability

Data are unavailable due to privacy or ethical restrictions.
